# The Progress of Immunotherapy in Refractory Pituitary Adenomas and Pituitary Carcinomas

**DOI:** 10.3389/fendo.2020.608422

**Published:** 2020-12-11

**Authors:** Congxin Dai, Siyu Liang, Bowen Sun, Jun Kang

**Affiliations:** ^1^ Department of Neurosurgery, Beijing Tongren Hospital, Capital Medical University, Beijing, China; ^2^ Eight-Year Program of Clinical Medicine, Peking Union Medical College Hospital (PUMCH), Chinese Academe of Medical Sciences & Peking Union Medical College (CAMS & PUMC), Beijing, China

**Keywords:** pituitary carcinomas, refractory pituitary adenomas, immunotherapy, programmed death-ligand 1, tumor-infiltrating lymphocytes

## Abstract

Most pituitary adenomas (PAs) are considered benign tumors, but approximately 0.2% can present metastasis and are classified as pituitary carcinomas (PCs). Refractory PAs lie between benign adenomas and true malignant PC and are defined as aggressive-invasive PAs characterized by a high Ki-67 index, rapid growth, frequent recurrence, and resistance to conventional treatments, including temozolomide. It is notoriously difficult to manage refractory PAs and PC because of the limited therapeutic options. As a promising therapeutic approach, cancer immunotherapy has been experimentally used for the treatment of many tumors, including pituitary tumors. The purpose of this review is to report the progress of immunotherapy in pituitary tumors, including refractory PAs and PCs. The tumor immune microenvironment has been recognized as a key contributor to tumorigenesis, progression, and prognosis. One study indicated that the number of CD68+ macrophages was positively correlated with tumor size and Knosp classification grade for tumor invasiveness. The infiltration of CD4+ and CD8+ T cells was relatively scant in these adenomas, but pituitary growth hormone (GH) adenomas exhibited significantly more CD4+ and CD8+ T cells than non-GH adenomas. These results suggest an association of CD68+ macrophage infiltration with an increase in pituitary tumor size and invasiveness. Another study suggested that a lower number of CD8+ lymphocytes is associated with cavernous sinus invasion and resistance to treatment with first-generation somatostatin analogs in acromegaly patients, highlighting a potential role of the tumor immune microenvironment in determining the prognosis of somatotroph pituitary tumors. Preclinical studies have indicated that widely varying degrees of programmed death-ligand 1 (PD-L1) expression and tumor-infiltrating lymphocytes (TILs) are found among different subtypes. Functional PAs and aggressive PAs express significantly higher levels of PD-L1 and TILs than other subtypes, indicating that PD-1 blockade might be a promising alternative therapy for patients with aggressive PAs. PD-L1 transcript and protein levels were found to be significantly increased in functioning (GH and prolactin-expressing) pituitary tumors compared to nonfunctioning (null cell and silent gonadotroph) adenomas. Moreover, primary pituitary tumors harbored higher levels of PD-L1 mRNA than recurrent tumors. These findings suggest the possibility of considering checkpoint blockade immunotherapy for functioning pituitary tumors refractory to conventional management. Animal models of Cushing’s disease also demonstrated PD-L1 and TIL expression in cultured tumors and murine models, as well as the effectiveness of checkpoint blockade therapy in reducing the tumor mass, decreasing hormone secretion, and increasing the survival rate. Clinical studies show that immunotherapy may be an effective treatment in patients with pituitary tumors. One corticotroph carcinoma patient showed a significant reduction in hormone levels and shrinkage of the tumor size of primary and metastatic lesions immediately after investigational treatment with ipilimumab and nivolumab. However, another patient with corticotroph adenoma progressed rapidly after four cycles of anti-PD-1 (pembrolizumab) treatment. To date, there are two registered clinical trials of immunotherapy for pituitary tumors. One of them is the phase II clinical trial of nivolumab combined with ipilimumab for patients with aggressive pituitary tumors (NCT04042753). The other one is also a phase II clinical trial of the combination of nivolumab and ipilimumab for rare tumors, including pituitary tumors (NCT02834013). Both clinical trials are in the stage of recruiting patients and have not been completed. In summary, the results from preclinical research and clinical studies indicated that immunotherapy might be a promising alternative therapy for PCs and refractory PAs resistant to conventional treatments. The combination of immunotherapy and radiotherapy or temozolomide may have synergistic effects compared to a single treatment. More preclinical and clinical studies are needed to further indicate the exact efficacy of immunotherapy in pituitary tumors.

## Introduction

Pituitary tumors represent approximately 10%–15% of intracranial tumors and are the second most common primary brain tumor in humans (1, 2). Most pituitary tumors are noninvasive benign tumors that grow slowly and remain within the sella and/or displace the surrounding tissues. However, up to 35% of them are invasive adenomas and infiltrate adjacent tissues, including the cavernous sinuses, bone, sphenoid sinuses, and nerve sheaths (3). Approximately 10% of pituitary adenomas (PAs) show aggressive clinical behavior and are refractory to conventional therapy (4–6). According to the current WHO classification (2017), aggressive PAs were defined in patients with a radiologically invasive tumor and unusually rapid tumor growth rate, or clinically relevant tumor growth despite optimal standard therapies (surgery, radiotherapy, and medical treatments) (5). Pituitary carcinomas (PCs) are defined as tumors of adenohypophyseal origin that show metastatic spread by either craniospinal dissemination or systemic metastases; PCs are very rare and represent only 0.1%–0.2% of all pituitary tumors (7–9). Although the understanding of the molecular pathogenesis of PCs has progressed rapidly, the early identification of PC is difficult until the presence of metastatic lesions. Refractory PAs lie between benign adenomas and true malignant PC and are defined as aggressive-invasive PAs characterized by a high Ki-67 index, rapid growth, frequent recurrence, and resistance to conventional treatments, including temozolomide (TMZ) (10, 11). Therefore, both refractory PAs and PCs are very difficult to manage due to the lack of effective treatment, and they are associated with increased morbidity and mortality (12).

Recently, TMZ has shown promising efficacy for pituitary tumors and has been recommended as a first-line medication for refractory PAs and PCs by the European Society of Endocrinology (5). However, previous studies showed that only approximately 60% of pituitary tumors are responsive to TMZ treatment, and some of them are resistant to TMZ (13, 14). Emerging targeted therapies, including those targeting vascular endothelial growth factor, epidermal growth factor, fibroblast growth factor, the Raf/Mek/ErK pathway, the PI3K/Akt/mTOR pathway, the notch signaling pathway, the hedgehog signaling pathway, and CDK 4/6, have been studied preclinically and/or have been experimentally used for refractory PAs and PCs, but their efficacy is limited (15–17). Therefore, more effective and novel treatment approaches are needed. As a promising therapeutic approach, cancer immunotherapy has been experimentally used for the treatment of many tumors, including pituitary tumors, and has been proposed as a potential treatment option for refractory PAs and PCs. The purpose of this review is to summarize the recent advances of immunotherapy in pituitary tumors, including refractory PAs and PCs.

## Mechanisms of Immunotherapy

The human immune system is responsible for discriminating self from non-self, thereby protecting the body from tumors recognized as non-self or altered self. The immune system implements surveillance against cancer by generating innate and adaptive immune responses against tumor antigens. Early efforts to strengthen the immune response provide a critical foundation for tumor immunotherapy, despite the products of this era, such as tumor necrosis factor and interleukin-2, being no longer used routinely for tumors due to unacceptable systemic side effects (18). Immune responses are capable of suppressing tumor growth and achieving tumor regression; nevertheless, tumors can evolve to evade immune system elimination for further proliferation, infiltration, and metastasis. The upregulation of T cell coinhibitory ligands plays a key role in tumor immune escape and provides a solid basis for the clinical evaluation of therapeutic strategies that target immune checkpoints (19, 20).

Immune checkpoints, the negative regulators of immune activation, are essential for controlling the strength of the immune response, maintaining self-tolerance, and reducing tissue damage. Tumors exploit immune checkpoints negatively regulate the activation of T cells. The blockade of the coinhibitory cytotoxic T-lymphocyte–associated protein 4 (CTLA-4) and programmed cell death 1 (PD-1) allows the activation of T cell stimulatory signaling, thereby enhancing antitumor T cell cytotoxicity, proinﬂammatory cytokine production, and proliferation and promoting tumor destruction (21–24).

To date, several monoclonal antibodies that block immune checkpoints have been developed and have achieved medium to high response rate. However, checkpoint inhibitors have not been approved for the treatment of PAs and PCs by the Food and Drug Administration (FDA). Interestingly, patients treated with checkpoint inhibitors can develop severe hypophysitis (25–27). This immune-related adverse event (irAE) provides compelling evidence that checkpoint blockade stimulates an immune response readily within the pituitary gland. However, checkpoint inhibitors are not effective for all; therefore, a variety of directions have emerged in current immunotherapy. The combination of CTLA-4 and PD-1/PD-L1 has achieved higher clinical remission due to the nonredundant coinhibitory roles of these two pathways, yet with a higher frequency of immune-related toxicities (28). Although usually well tolerated, combination therapy increases the risk of high-grade irAEs. The establishment of clinical biomarkers helps to identify patients who will beneﬁt from checkpoint inhibitors and guide individualized treatment with optimal doses. It has demonstrated that immune checkpoint inhibitor efficacy is affected by a combination of factors involving tumor genomics, host germline genetics, PD1 ligand 1 (PDL1) levels, and other features of the tumor microenvironment, as well as the gut microbiome (29). Several possible candidates, including tumor-infiltrating T lymphocyte quantification, immunostaining-based PD-L1, and sequencing-based mutational burden and neoantigen burden biomarkers, have been proposed for further evaluation (29, 30).

## Tumor Immune Microenvironment of Pituitary Tumors

**Figure 1:**
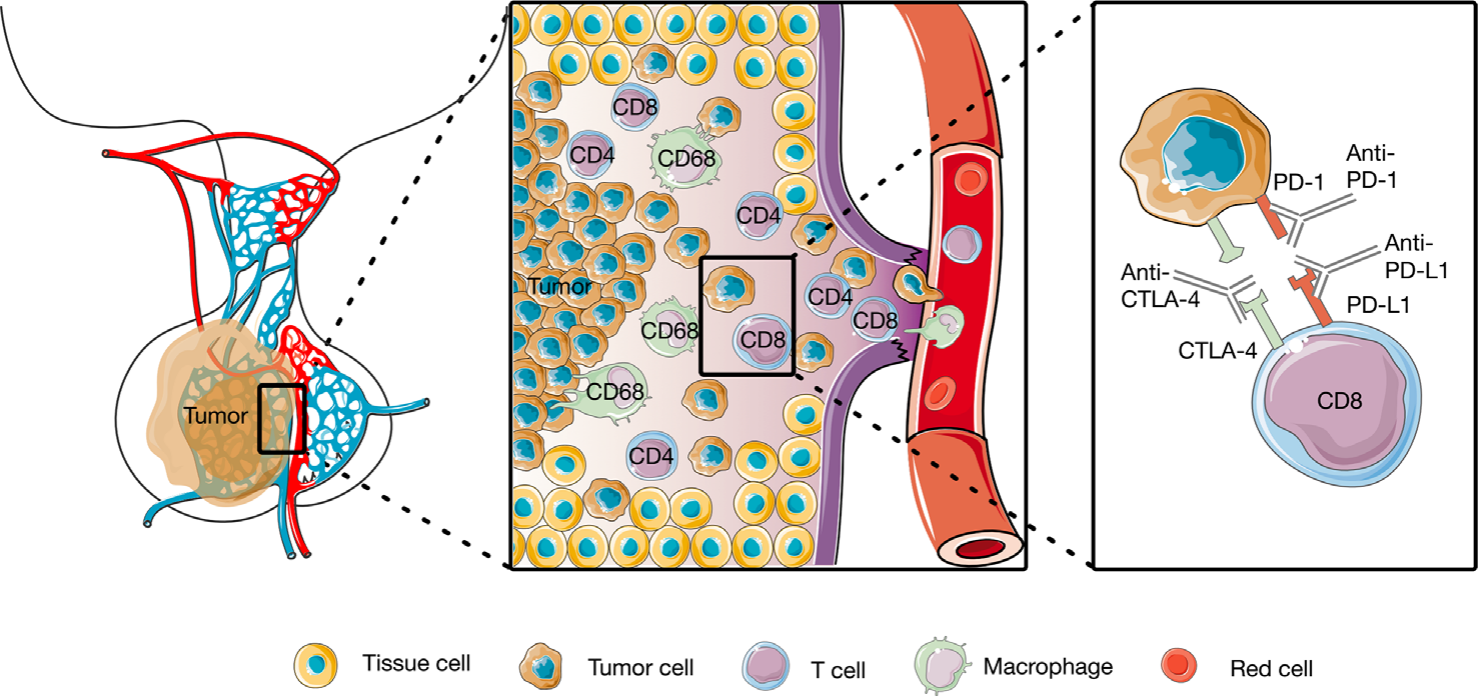
Tumor immune microenvironment of pituitary tumors and of Working model of anti-PD1 and anti-anti-CTLA4. The CD4+, CD8+, and CD68+ cells were identified in pituitary tumors, and anti-PD1 and anti-anti-CTLA4 rescue T cell anti-tumor effector functions.

The tumor immune microenvironment (TIM) plays a critical role in tumorigenesis and progression. It has demonstrated that certain features of the degree of tumor infiltration by cytotoxic T cells can predict clinical outcome of patients (31). Tumor-infiltrating lymphocytes (TILs) and other mononuclear cells, such as tumor-associated macrophages, are central components of the TIM, participate, and regulate the tumor immune response and have been used as prognostic factors in numerous solid tumors, such as melanomas and ovarian, breast, colorectal, and urothelial carcinomas (31–38). It has been possible to identify different subclasses of immune environment that have an influence on tumor initiation and response and therapy (37). Although PAs are a heterogeneous group of lesions with different clinical behaviors, TILs were found to be present in all subtypes of PAs, albeit at low levels (**Figure 1**) (39–43). The penetration of more than 5% CD8+ cells was observed in 66 of 191 PA patients (44). A retrospective study defined lymphocyte infiltration as the presence of 15 to 20 lymphocytes in the 400 times magnified field. They reported a TIL prevalence of 2.9% in all 1,400 PAs, including both functional and nonfunctional types (41). A case-control study scored CD45 expression on a scale ranging from 0 to 5 to obtain a semiquantitative measure of lymphocyte infiltration. The infiltration of CD45+ cells was mild, with a score of 1 or 0.5 in the majority of PAs, TILs are more abundant in adenoma patients than in healthy controls (40). TILs were first quantitated in 35 cases by counting the numbers of macrophages and lymphocytes in 10 to 30 consecutive high-power fields (HPFs) at 400× magnification. CD68+ macrophages were sparse, with an average number of 4–8 per HPF, and CD4+ and CD8+ cells were relatively rare, with an average number of lower than 4 per HPF in different secretory subtypes (42).

It is unclear whether the features of PAs are associated with immune cell infiltration. CD45+ infiltration was similar among different subtypes of PAs (40, 41). However, CD4+ and CD8+ staining was closely correlated with higher growth hormone (GH) levels (42–44). CD68 expression was higher in sparsely granulated GH adenomas and null cell adenomas than in densely granulated GH-secreting adenomas and adrenocorticotropic hormone (ACTH)-secreting adenomas (42).

The phenotype and functional consequences of TILs are complex; however, the overall presence of TILs and other mononuclear cells is associated with a high risk of recurrence and poor prognosis in patients with PAs. After a mean follow-up period of 34 ± 2 months, a poor clinical outcome was more common in PA patients with CD45 immunostaining than in those without CD45 immunostaining. The odds of a poor clinical outcome were 3.3-fold greater than that without CD45+ cells observed in adenomas (40). This may be due to the correlation between positive CD45 staining and a higher proliferative index. No correlations were found between CD8+ T cells and tumor size (42, 44) or Knosp classification grade (42). One study showed that the number of CD8+ lymphocytes tended to be higher in the cavernous sinus invasion group than in the cavernous sinus non-invasion group, but no significant difference was observed (45). The number of CD68+ cells was positively correlated with tumor size and Knosp classification grade for tumor invasiveness (42). An increased macrophage (CD68+ cells) infiltration was also observed in aryl hydrocarbon receptor interacting protein (AIP)-mutated PAs and was associated with the invasiveness of AIP-mutated adenomas (46). Together, these TILs and other mononuclear cells not only are related to the tumor characteristics and progression of pituitary tumors, but also offer for both prognosis and targeted therapeutics (47).

### PD-1/PD-L1 Expression in Pituitary Tumors

PD-L1 expression is observed to varying degrees in different subtypes (43–45, 48–50). PD-L1 is expressed on both the cell membrane and cytoplasm of tumor cells and occasionally in endothelial cells (45). PD-L1 staining is mild to moderate in most cases (43). One study found that in 36.6% of 191 cases, PD-L1 expression was observed in more than 5% of tumor cells (44), but another study detected the same PD-L1 levels in only 7.9% of 139 cases (48). The concordance of PD-L1 immunostaining between assays may be poor.

The tumor features affected by PD-L1 expression remain controversial. One study showed that PD-L1 expression significantly increased with elevated levels of serum GH, PRL, ACTH and cortisol (44). Consistent with their finding, others further found significantly higher PD-L1 expression in adenomas with clinical functioning and a high Ki-67 or MIB-1 proliferative index (43, 44, 51). Another study revealed an increased tendency of cavernous sinus invasion with higher PD-L1 expression (45). In addition, PD-L1 expression is positively correlated with increased TILs (43, 44). However, a retrospective series of 139 cases found that none of these features, including hormone secretion, aggressiveness, and invasiveness, correlated with PD-L1 expression (48). The findings of this study are inconsistent with those of previous studies, indicating that PD-L1 expression should be further studied among the diverse biological characteristics or behaviors of pituitary tumors.

The PD-L1 RNA transcript is significantly increased not only in GH-secreting and PRL-secreting adenomas compared to null cell and silent gonadotroph adenomas but also in primary pituitary tumors compared to recurrent tumors (43). However, the correlation between PD-L1 mRNA and protein levels was poor at the individual sample level in this study. In nonfunctioning PAs, PD-L1 mRNA transcription was signiﬁcantly higher in gonadotroph adenomas than in null cell and silent corticotroph adenomas and was accompanied by a more than 3% MIB-1 proliferative index (49). This finding suggests that PD-L1 mRNA levels are associated with aggressiveness in nonfunctioning PAs, but PD-L1 expression was not examined in this study. Although data are limited, it is possible that such tumor features inﬂuence PD-L1 expression levels, potentially aﬀecting checkpoint inhibitor eﬀectiveness.

The upregulated coinhibitory ligands, represented by PD-L1, indicate that PAs can evade immune surveillance through the suppression of the immune response (52–54). Functional PAs and aggressive PAs may express higher levels of PD-L1 than other subtypes, indicating that PD-1 blockade might be a promising alternative therapy for patients with aggressive PAs. These findings suggest the possibility of considering checkpoint blockade immunotherapy for pituitary tumors that resist conventional management.

## Preclinical Models of Pituitary Tumors

The establishment of preclinical models helps to improve our understanding of the mechanism of immunotherapy in PA patients. PD-L1 is prominently expressed in a murine pituitary ACTH adenoma cell line *in vitro*, second only to a melanoma cell line, and higher than that in breast adenocarcinoma, lung carcinoma, and glioma cell lines (51). This pattern of PD-L1 expression is still maintained *in vivo* after 8 weeks of growth in tumor implantation. Moreover, TILs are present in cultured tumors and have been confirmed to have a high expression of PD-1, TIM-3, and LAG-3, which marked the upregulation of coinhibitory molecules and may further cause TIL exhaustion. It has shown that the effect of anti PD-L1 on the preclinical model is only partially dependent on the T cells function. On the contrary, it seems that CD11b+ myeloid cells, which have high PD-L1 expression, may play a more important role in the response to anti-PD-L1 treatment. Thus, this finding will suggest that interaction between CD11b+ myeloid cells and cancer cells are critical for the survival of cancer cells, further reenforce the notion that myeloid cells are important therapeutic target (51). These findings of preclinical results provided important information for human tumors. However, there are some essential differences between these preclinical models and human tumors, more research related to human tumors are needed.

Given the presence of lymphocytes in PAs and the expression of coinhibitory ligands in the tumor microenvironment, PAs may be sensitive to checkpoint inhibitors. The efficacy of anti-PD-L1 antibodies has been examined in both subcutaneous and intracranial murine models of Cushing disease (51). Following subcutaneous tumor implantation, mice were treated intraperitoneally with either anti-PD-L1 antibody or isotype control antibody every 3 days for a total of 12 doses in 36 days. After 8 weeks, anti-PD-L1 treatment significantly inhibited tumor growth and suppressed serum ACTH secretion compared with untreated tumor-bearing mice. Some mice achieved complete tumor regression. To study the therapeutic capacity of anti-PD-L1 on intracranial lesions, the tumor was placed in the right frontal lobe, where the tumor uncontrollably grows, resulting in 100% mortality after approximately 3 weeks. Following intracranial tumor implantation, mice were given the same treatment as above every 3 days for a total of 15 doses or until the endpoint. The median overall survival was extended to 29.5 days in the anti-PD-L1 treatment group compared with a median survival of 21.5 days in the control group. Additionally, anti-PD-L1 therapy can improve long-term survival. In the treatment group, the 30-day survival rate was estimated to be 50%, and the 60-day survival rate was estimated to be 40%, while in the control group, the survival rate was 0% at both time points.

Preclinical models of Cushing disease demonstrate PD-L1 expression in cell lines and cultured tumors as well as the effectiveness of anti-PD-L1 immunotherapy in reducing tumor mass, decreasing hormone secretion, and arresting tumor proliferation. Although only a few data points are available, murine and cell line studies indicate that PD-L1 is a potential target in PAs.

## Clinical Studies of Immunotherapy in Pituitary Tumors

Pituitary tumors are usually benign and slow growing, but a subset has a more aggressive clinical behavior. PCs, defined by the presence of cerebrospinal or distant metastasis of a pituitary neuroendocrine tumor, are particularly rare and have mortality rates of up to 66% at 1 year after diagnosis. For many patients with refractory PAs and PCs, effective targeted therapy is still lacking (17). PCs have a very limited response to previous therapies, including somatostatin analogs, external beam radiotherapy, chemotherapies including TMZ, capecitabine, everolimus, sunitinib, and bevacizumab, and peptide receptor radionuclide therapy (55). TMZ has been established as a first-line chemotherapeutic treatment for aggressive PAs or PCs. However, in a large cohort including 157 patients treated with TMZ, complete response was observed in only a median of 6% of patients after a median treatment period of 9 cycles. Thirty-one percent achieved partial response, 33% achieved disease stabilization, but 33% had disease progression (56). Of note, of the patients who had complete response, partial response and stable disease, 25%, 40%, and 48%, respectively, further progressed after a median of 12 months of follow-up. The limited long-term effect of TMZ highlights the need to identify additional effective therapies.

The increased understanding of the TIM has signiﬁcantly improved the effort to enhance the immune response and has brought about advances in cancer therapies over recent years. Similarly, recent data have shown the expression of PD-L1 and the presence of TILs in PA, as well as the possible association of the immune microenvironment with the biological and clinical phenotypes of PA, including hormone secretion, invasiveness, and aggressiveness. These findings provide a rationale to attempt the use of checkpoint inhibitors in some clinical scenarios, although strong support for the use of checkpoint inhibitors in PA is still lacking.

It has been proved that PD1/PDL1 blockade significantly rescue T cell anti-tumor effector functions by interfering with T cell activation and the acquisition of effector capacities (57). The CTLA-4 is a surface molecule expressed by activated T cells, inhibition of CTLA-4 may increase the regulation of the immune response to cancer cells (58). Combination therapy with anti-CTLA4 plus anti-PD1 monocolonal antibodies not only leads to an increased frequency of ICOS+ CD4+ effector T cells, but also leads to unexpected and unique changes including a decreased frequency of exhausted CD8+ T cells and the expansion of activated CD8+ effector T cells (24). Only one patient with ACTH-secreting PC has been reported to be successfully treated with immunotherapy until now (59). A 35-year-old woman with aggressive ACTH-secreting PA received medical therapies, including pasireotide, ketoconazole, and ketoconazole combined with cabergoline, followed by several surgical resections and radiation therapies due to tumor enlargement and incomplete hormonal control. Afterwards, she was treated with TMZ and capecitabine but discontinued after four cycles due to poor tolerance, despite the decrease in tumor volume and ACTH level observed during this period. Sixty-eight months after the initial diagnosis, the patient presented with liver metastatic lesions. Disease progressed intracranially and extracranially during two additional cycles of TMZ combined with capecitabine. She then started immunotherapy with an anti-CTLA-4 agent (ipilimumab) combined with an anti-PD-1 antibody (nivolumab). Following five cycles, the patient yielded a 92% regression in the dominant hepatic metastasis, a 59% decrease in the recurrent intracranial lesions, and a normalization in plasma ACTH levels to 66 pg/ml from the previous 45,550 pg/ml, with acceptable drug side effects. It is worth noting that subsequent analysis of the hepatic metastasis demonstrated an MSH6 mutation and 1% PD-L1 expression. This case revealed that checkpoint inhibition should be a treatment consideration for refractory PAs and PCs, especially for tumors that have developed resistance to TMZ. It was supposed that TMZ-induced hypermutated tumors may be more sensitive to checkpoint inhibitors. TMZ can induce alterations in the mismatch repair system and, consequently, generate a greater number of neoantigens, which results in a greater efficacy of treatment with checkpoint inhibitors (60).

However, the generalizability of checkpoint inhibitors needs further study. Another case reported that pembrolizumab had poor efficacy in the treatment of refractory ACTH-secreting PA (61). A 47-year-old man had partial response to TMZ for the initial 6 weeks, and then treatment was changed to pasireotide monotherapy until disease progression. Further immunohistochemical analysis revealed a complete loss of MSH2 and MSH6. Notably, no PD-L1 expression was found in PA immunostaining. The patient then received immunotherapy with an anti-PD-1 antibody (pembrolizumab). After four cycles, the patient showed radiological progression of disease and an increase in serum ACTH and urinary cortisol levels. The failure may be explained by the weakened efficacy of anti-PD-L1 and the decreased immune response, which may result from the consistently high levels of serum cortisol in tumors. Further investigation is needed to find reliable biomarkers to predict the response to immunotherapy. Although many studies have explored measurements of PD-L1 expression as a predictor of response to checkpoint inhibitors, it remains primarily a research tool. In current clinical practice, PD-L1 expression is limited as a predictor of the response to immunotherapy. PD-L1 staining may not be used to accurately select patients for PD-1/PD-L1 pathway blockade due to the low prediction accuracy and dynamic changes (62). In some studies, the expression of PD-L1 appeared to strongly predict the efficacy of anti-PD-L1, whereas in other studies, poor predictive value has been found. Although the immune checkpoint inhibitors have shown good efficacy in some cases, anti-PD1 alone may not be potent enough. A strategy to combine agent targeting the immunosuppressive TME with immune checkpoint blockade may overcome the limitation.

Immunotherapy appears to have some clinical beneﬁt in patients with PAs and may be an option for medical therapy in refractory PAs and PCs. Nevertheless, the limitations of immunotherapy should be acknowledged, as the clinical response is variable and predictive efficacy is challenging. Further studies are still needed to identify the optimal use of this treatment in different clinical scenarios.

## Future Perspectives

### The Need for Further Improvement of Potential Biomarkers

Although no immunotherapy for PAs has been approved yet, various studies have been carried out to explore immune-related biomarkers or therapeutic targets. In addition to PD-L1 and TILs, which have been detailed above, other important aspects have also been investigated. A range of transcriptomics analyses have been conducted to identify genes expressed differentially between PA and normal samples (63–66). However, high inconsistency was found among different analyses because of the limited number of both the patients and controls in each analysis. Using a novel strategy of data integration, one bioinformatics study integrated available PA microarray datasets to identify more robust differentially expressed genes and to annotate immune-related genes (67). Based on the analysis of the human protein-protein interaction network, some promising target candidates, including GAL, LMO4, STAT3, PD-L1, TGFB, and TGFBR3, were proposed for PA immunotherapy. This study provides useful guidance for the development of novel biomarkers for PA immunotherapy. To better understand the composition of the TIM of pituitary tumors, more novel technologies like single cell RNA-seq and imaging mass cytometry are needed for new immunotherapy development.

### The Need for the Standardization of Assessment Methods and Verification in Animal Models

Previous studies used different antibody clones and cut-off values (48). The lack of consensus in assessment criteria partly limited studies on PD-L1 and other ligands as markers of response to checkpoint inhibitors in patients with refractory or TMZ unresponsive PAs and PCs. Therefore, the standardization of methodologies to verify the prognostic value in a large cohort is a prerequisite for the future routine application of immunotherapy (68).

Over 40 animal models for PAs have been generated. Many of these models can represent human syndromes (69). Some models can even simulate drug resistance (70). Establishment and use of patient-derived xenograft models has been widely used for drug testing in many cancers (71), which may offer a perfect mouse model for immunotherapy of pituitary tumors. Evaluation in these models will increase our understanding of the immune microenvironment in PAs and of the roles of immunotherapy.

### The Need for Prospective Clinical Studies on Immunotherapy

Case reports revealed that checkpoint inhibitors represent a possible therapy for PAs and adenocarcinoma. Further prospective clinical studies are warranted. To date, there are two registered clinical trials of immunotherapy for pituitary tumors (72, 73). One of them is the phase II clinical trial entitled Nivolumab and Ipilimumab in People with Aggressive Pituitary Tumors (Memorial Sloan Kettering Cancer Center, United States, NCT04042753). The other one is also a phase II clinical trial called Nivolumab and Ipilimumab in Treating Patients with Rare Tumors (National Cancer Institute, United States, NCT02834013), which includes pituitary tumors. Both clinical trials are in the stage of recruiting patients and have not been completed.

## Conclusion

In summary, it is notoriously difficult to manage refractory PAs and PCs due to the limited effective therapeutic options. As a promising therapeutic approach, cancer immunotherapy in pituitary tumors has recently attracted increasing attention. The TIM has been recognized as a key contributor to the tumorigenesis, progression, invasion, and prognosis of pituitary tumors. The expression of macrophages, lymphocytes and PD-L1 varied greatly in different pituitary tumors, and these immune factors are associated with the clinicopathological characteristics of pituitary tumors. Clinical case studies show that immunotherapy appears to have some clinical benefit in patients with refractory PAs or PCs. However, although these data suggest that cancer immunotherapy may be an effective therapeutic target for patients with refractory PAs and PCs, further basic research and clinical trials are needed to verify these findings.

## Author Contributions

All authors listed have made a substantial, direct, and intellectual contribution to the work, and approved it for publication.

## Funding

The financial support for this study was provided by the Scientific Research Project of Capital Health Development in 2020 (grant number: 2020-2-2058), and Beijing Natural Science Foundation (grant number: 7172057). The funding institutions had no role in the design of the study, data collection and analysis, the decision to publish, or the preparation of the manuscript.

## Conflict of Interest

The authors declare that the research was conducted in the absence of any commercial or financial relationships that could be construed as a potential conflict of interest.

## References

[1] Molitch ME Diagnosis and Treatment of Pituitary Adenomas: A Review. JAMA (2017) 317(5):516–24. 10.1001/jama.2016.19699 28170483

[2] Melmed S Pathogenesis of pituitary tumors. Nat Rev Endocrinol (2011) 7(5):257–66. 10.1038/nrendo.2011.40 21423242

[3] Hansen TM Batra S Lim M Gallia GL Burger PC Salvatori R Invasive adenoma and pituitary carcinoma: a SEER database analysis. Neurosurg Rev (2014) 37(2):279–85; discussion 85-6. 10.1007/s10143-014-0525-y PMC4322934 24526366

[4] Kasuki L Raverot G Definition and diagnosis of aggressive pituitary tumors. Rev Endocr Metab Disord (2020) 21(2):203–8. 10.1007/s11154-019-09531-x 31808044

[5] Raverot G Burman P McCormack A Heaney A Petersenn S Popovic V European Society of Endocrinology Clinical Practice Guidelines for the management of aggressive pituitary tumours and carcinomas. Eur J Endocrinol (2018) 178(1):G1–G24. 10.1530/EJE-17-0796 29046323

[6] Lopes MBS The 2017 World Health Organization classification of tumors of the pituitary gland: a summary. Acta Neuropathol (2017) 134(4):521–35. 10.1007/s00401-017-1769-8 28821944

[7] Kaltsas GA Nomikos P Kontogeorgos G Buchfelder M Grossman AB Clinical review: Diagnosis and management of pituitary carcinomas. J Clin Endocrinol Metab (2005) 90(5):3089–99. 10.1210/jc.2004-2231 15741248

[8] Daly AF Tichomirowa MA Beckers A The epidemiology and genetics of pituitary adenomas. Best Pract Res Clin Endocrinol Metab (2009) 23(5):543–54. 10.1016/j.beem.2009.05.008 19945022

[9] Heaney AP Clinical review: Pituitary carcinoma: difficult diagnosis and treatment. J Clin Endocrinol Metab (2011) 96(12):3649–60. 10.1210/jc.2011-2031 PMC3277423 21956419

[10] Dai C Liu X Ma W Wang R The Treatment of Refractory Pituitary Adenomas. Front Endocrinol (Lausanne) (2019) 10:334. 10.3389/fendo.2019.00334 31191457 PMC6548863

[11] Syro LV Rotondo F Camargo M Ortiz LD Serna CA Kovacs K Temozolomide and Pituitary Tumors: Current Understanding, Unresolved Issues, and Future Directions. Front Endocrinol (Lausanne) (2018) 9:318. 10.3389/fendo.2018.00318 29963012 PMC6013558

[12] Ilie MD Jouanneau E Raverot G Aggressive Pituitary Adenomas and Carcinomas. Endocrinol Metab Clin North Am (2020) 49(3):505–15. 10.1016/j.ecl.2020.05.008 32741485

[13] Ji Y Vogel RI Lou E Temozolomide treatment of pituitary carcinomas and atypical adenomas: systematic review of case reports. Neurooncol Pract (2016) 3(3):188–95. 10.1093/nop/npv059 PMC4986928 27551432

[14] McCormack AI Wass JA Grossman AB Aggressive pituitary tumours: the role of temozolomide and the assessment of MGMT status. Eur J Clin Invest (2011) 41(10):1133–48. 10.1111/j.1365-2362.2011.02520.x 21496012

[15] Araki T Cooper O Fukuoka H Editorial: Targeted Therapy for Pituitary Adenomas. Front Endocrinol (Lausanne) (2019) 10:358. 10.3389/fendo.2019.00358 31214126 PMC6557996

[16] Dai C Zhang B Liu X Ma S Yang Y Yao Y Inhibition of PI3K/AKT/mTOR pathway enhances temozolomide-induced cytotoxicity in pituitary adenoma cell lines in vitro and xenografted pituitary adenoma in female nude mice. Endocrinology (2013) 154(3):1247–59. 10.1210/en.2012-1908 23384836

[17] Lamb LS Sim H-W McCormack AI Exploring the Role of Novel Medical Therapies for Aggressive Pituitary Tumors: A Review of the Literature-“Are We There Yet?”. Cancers (Basel) (2020) 12(2):E308. 10.3390/cancers12020308 32012988 PMC7072681

[18] Abbott M Ustoyev Y Cancer and the Immune System: The History and Background of Immunotherapy. Semin Oncol Nurs (2019) 35(5):150923. 10.1016/j.soncn.2019.08.002 31526550

[19] O’Donnell JS Teng MWL Smyth MJ Cancer immunoediting and resistance to T cell-based immunotherapy. Nat Rev Clin Oncol (2019) 16(3):151–67. 10.1038/s41571-018-0142-8 30523282

[20] Corrales L Matson V Flood B Spranger S Gajewski TF Innate immune signaling and regulation in cancer immunotherapy. Cell Res (2017) 27(1):96–108. 10.1038/cr.2016.149 27981969 PMC5223230

[21] Ribas A Wolchok JD Cancer immunotherapy using checkpoint blockade. Science (2018) 359(6382):1350–5. 10.1126/science.aar4060 PMC7391259 29567705

[22] McLane LM Abdel-Hakeem MS Wherry EJ CD8 T Cell Exhaustion During Chronic Viral Infection and Cancer. Annu Rev Immunol (2019) 37:457–95. 10.1146/annurev-immunol-041015-055318 30676822

[23] Baumeister SH Freeman GJ Dranoff G Sharpe AH Coinhibitory pathways in immunotherapy for cancer. Annu Rev Immunol (2016) 34:539–73. 10.1146/annurev-immunol-032414-112049 26927206

[24] Sharma P Allison JP Dissecting the mechanisms of immune checkpoint therapy. Nat Rev Immunol (2020) 20(2):75–6. 10.1038/s41577-020-0275-8 31925406

[25] Atkins P Ur E Primary and Ipilimumab-induced Hypophysitis: A Single-center Case Series. Endocr Res (2020) 45(4):246–53. 10.1080/07435800.2020.1817064 32892666

[26] Faje A Immunotherapy and hypophysitis: clinical presentation, treatment, and biologic insights. Pituitary (2016) 19(1):82–92. 10.1007/s11102-015-0671-4 26186958

[27] Kennedy LB Salama AKS A review of cancer immunotherapy toxicity. CA Cancer J Clin (2020) 70(2):86–104. 10.3322/caac.21596 31944278

[28] Larkin J Chiarion-Sileni V Gonzalez R Grob JJ Cowey CL Lao CD Combined Nivolumab and Ipilimumab or Monotherapy in Untreated Melanoma. N Engl J Med (2015) 373(1):23–34. 10.1056/NEJMoa1504030 26027431 PMC5698905

[29] Havel JJ Chowell D Chan TA The evolving landscape of biomarkers for checkpoint inhibitor immunotherapy. Nat Rev Cancer (2019) 19(3):133–50. 10.1038/s41568-019-0116-x PMC6705396 30755690

[30] Gibney GT Weiner LM Atkins MB Predictive biomarkers for checkpoint inhibitor-based immunotherapy. Lancet Oncol (2016) 17(12):e542–e51. 10.1016/S1470-2045(16)30406-5 PMC5702534 27924752

[31] Giraldo NA Sanchez-Salas R Peske JD Vano Y Becht E Petitprez F The clinical role of the TME in solid cancer. Br J Cancer (2019) 120(1):45–53. 10.1038/s41416-018-0327-z 30413828 PMC6325164

[32] Clemente CG Mihm MC Jr. Bufalino R Zurrida S Collini P Cascinelli N Prognostic value of tumor infiltrating lymphocytes in the vertical growth phase of primary cutaneous melanoma. Cancer (1996) 77(7):1303–10. 10.1002/(SICI)1097-0142(19960401)77:7<1303::AID-CNCR12>3.0.CO;2-5 8608507

[33] Sato E Olson SH Ahn J Bundy B Nishikawa H Qian F Intraepithelial CD8+ tumor-infiltrating lymphocytes and a high CD8+/regulatory T cell ratio are associated with favorable prognosis in ovarian cancer. Proc Natl Acad Sci U S A (2005) 102(51):18538–43. 10.1073/pnas.0509182102 PMC1311741 16344461

[34] Smyrk TC Watson P Kaul K Lynch HT Tumor-infiltrating lymphocytes are a marker for microsatellite instability in colorectal carcinoma. Cancer (2001) 91(12):2417–22. 10.1002/1097-0142(20010615)91:12<2417::aid-cncr1276>3.0.co;2-u 11413533

[35] Sharma P Shen Y Wen S Yamada S Jungbluth AA Gnjatic S CD8 tumor-infiltrating lymphocytes are predictive of survival in muscle-invasive urothelial carcinoma. Proc Natl Acad Sci U S A (2007) 104(10):3967–72. 10.1073/pnas.0611618104 PMC1820692 17360461

[36] Zhang QW Liu L Gong CY Shi HS Zeng YH Wang XZ Prognostic significance of tumor-associated macrophages in solid tumor: a meta-analysis of the literature. PLoS One (2012) 7(12):e50946. 10.1371/journal.pone.0050946 23284651 PMC3532403

[37] Binnewies M Roberts EW Kersten K Chan V Fearon DF Merad M Understanding the tumor immune microenvironment (TIME) for effective therapy. Nat Med (2018) 24(5):541–50. 10.1038/s41591-018-0014-x PMC5998822 29686425

[38] Ilie MD Vasiljevic A Raverot G Bertolino P The Microenvironment of Pituitary Tumors-Biological and Therapeutic Implications. Cancers (Basel) (2019) 11(10):1605. 10.3390/cancers11101605 PMC6826349 31640258

[39] Rossi ML Jones NR Esiri MM Havas L Izzi M Coakham HB Mononuclear cell infiltrate and HLA-Dr expression in 28 pituitary adenomas. Tumori (1990) 76(6):543–7. 10.1177/030089169007600605 2284689

[40] Lupi I Manetti L Caturegli P Menicagli M Cosottini M Iannelli A Tumor infiltrating lymphocytes but not serum pituitary antibodies are associated with poor clinical outcome after surgery in patients with pituitary adenoma. J Clin Endocrinol Metab (2010) 95(1):289–96. 10.1210/jc.2009-1583 PMC2805498 19875479

[41] Heshmati HM Kujas M Casanova S Wollan PC Racadot J Van Effenterre R Prevalence of lymphocytic infiltrate in 1400 pituitary adenomas. Endocr J (1998) 45(3):357–61. 10.1507/endocrj.45.357 9790270

[42] Lu J-Q Adam B Jack AS Lam A Broad RW Chik CL Immune Cell Infiltrates in Pituitary Adenomas: More Macrophages in Larger Adenomas and More T Cells in Growth Hormone Adenomas. Endocr Pathol (2015) 26(3):263–72. 10.1007/s12022-015-9383-6 26187094

[43] Mei Y Bi WL Greenwald NF Du Z Agar NYR Kaiser UB Increased expression of programmed death ligand 1 (PD-L1) in human pituitary tumors. Oncotarget (2016) 7(47):76565–76. 10.18632/oncotarget.12088 PMC5363530 27655724

[44] Wang P-F Wang T-J Yang Y-K Yao K Li Z Li YM The expression profile of PD-L1 and CD8(+) lymphocyte in pituitary adenomas indicating for immunotherapy. J Neuro Oncol (2018) 139(1):89–95. 10.1007/s11060-018-2844-2 29680903

[45] Sato M Tamura R Tamura H Mase T Kosugi K Morimoto Y Analysis of Tumor Angiogenesis and Immune Microenvironment in Non-Functional Pituitary Endocrine Tumors. J Clin Med (2019) 8(5):695. 10.3390/jcm8050695 PMC6572068 31100921

[46] Barry S Carlsen E Marques P Stiles CE Gadaleta E Berney DM Tumor microenvironment defines the invasive phenotype of AIP-mutation-positive pituitary tumors. Oncogene (2019) 38(27):5381–95. 10.1038/s41388-019-0779-5 PMC6755983 30867568

[47] DeNardo DG Ruffell B Macrophages as regulators of tumour immunity and immunotherapy. Nat Rev Immunol (2019) 19(6):369–82. 10.1038/s41577-019-0127-6 PMC7339861 30718830

[48] Suteau V Collin A Menei P Rodien P Rousselet MC Briet C Expression of programmed death-ligand 1 (PD-L1) in human pituitary neuroendocrine tumor. Cancer Immunol Immunother (2020) 69(10):2053–61. 10.1007/s00262-020-02611-x PMC11027687 32445029

[49] Uraki S Ariyasu H Doi A Takeshima K Morita S Inaba H MSH6/2 and PD-L1 Expressions Are Associated with Tumor Growth and Invasiveness in Silent Pituitary Adenoma Subtypes. Int J Mol Sci (2020) 21(8):2831. 10.3390/ijms21082831 PMC7215962 32325698

[50] Salomon MP Wang X Marzese DM Hsu SC Nelson N Zhang X The epigenomic landscape of pituitary adenomas reveals specific alterations and differentiates among acromegaly, Cushing’s disease and endocrine-inactive subtypes. Clin Cancer Res (2018) 24(17):4126–36. 10.1158/1078-0432 30084836

[51] Kemeny HR Elsamadicy AA Farber SH Champion CD Lorrey SJ Chongsathidkiet P Targeting PD-L1 Initiates Effective Antitumor Immunity in a Murine Model of Cushing Disease. Clin Cancer Res (2020) 26(5):1141–51. 10.1158/1078-0432.CCR-18-3486 PMC7809696 31744830

[52] Kim YH Kim JH Transcriptome Analysis Identifies an Attenuated Local Immune Response in Invasive Nonfunctioning Pituitary Adenomas. Endocrinol Metab (Seoul) (2019) 34(3):314–22. 10.3803/EnM.2019.34.3.314 PMC6769343 31565884

[53] Srirangam Nadhamuni V Korbonits M Novel insights into Pituitary Tumorigenesis: Genetic and Epigenetic Mechanisms. Endocr Rev (2020) 41(6):bnaa006. 10.1210/endrev/bnaa006 32201880 PMC7441741

[54] Elsarrag M Patel PD Chatrath A Taylor D Jane JA Genomic and molecular characterization of pituitary adenoma pathogenesis: review and translational opportunities. Neurosurg Focus (2020) 48(6):E11. 10.3171/2020.3.FOCUS20104 32480367

[55] Alshaikh OM Asa SL Mete O Ezzat S An Institutional Experience of Tumor Progression to Pituitary Carcinoma in a 15-Year Cohort of 1055 Consecutive Pituitary Neuroendocrine Tumors. Endocr Pathol (2019) 30(2):118–27. 10.1007/s12022-019-9568-5 30706322

[56] McCormack A Dekkers OM Petersenn S Popovic V Trouillas J Raverot G Treatment of aggressive pituitary tumours and carcinomas: results of a European Society of Endocrinology (ESE) survey 2016. Eur J Endocrinol (2018) 178(3):265–76. 10.1530/EJE-17-0933 29330228

[57] Hui E Immune checkpoint inhibitors. J Cell Biol (2019) 218(3):740–1. 10.1083/jcb.201810035 PMC6400575 30760493

[58] de Miguel M Calvo E Clinical Challenges of Immune Checkpoint Inhibitors. Cancer Cell (2020) 38(3):326–33. 10.1016/j.ccell.2020.07.004 32750319

[59] Lin AL Jonsson P Tabar V Yang TJ Cuaron J Beal K Marked Response of a Hypermutated ACTH-Secreting Pituitary Carcinoma to Ipilimumab and Nivolumab. J Clin Endocrinol Metab (2018) 103(10):3925–30. 10.1210/jc.2018-01347 PMC6456994 30085142

[60] Cahill DP Levine KK Betensky RA Codd PJ Romany CA Reavie LB Loss of the mismatch repair protein MSH6 in human glioblastomas is associated with tumor progression during temozolomide treatment. Clin Cancer Res (2007) 13(7):2038–45. 10.1158/1078-0432.CCR-06-2149 PMC2873832 17404084

[61] Caccese M Barbot M Ceccato F Padovan M Gardiman MP Fassan M Rapid disease progression in patient with mismatch-repair deficiency pituitary ACTH-secreting adenoma treated with checkpoint inhibitor pembrolizumab. Anti Cancer Drugs (2020) 31(2):199–204. 10.1097/CAD.0000000000000856 31702999

[62] Meng X Huang Z Teng F Xing L Yu J Predictive biomarkers in PD-1/PD-L1 checkpoint blockade immunotherapy. Cancer Treat Rev (2015) 41(10):868–76. 10.1016/j.ctrv.2015.11.001 26589760

[63] Morris DG Musat M Czirjak S Hanzely Z Lillington DM Korbonits M Differential gene expression in pituitary adenomas by oligonucleotide array analysis. Eur J Endocrinol (2005) 153(1):143–51. 10.1530/eje.1.01937 15994756

[64] Tong Y Zheng Y Zhou J Oyesiku NM Koeffler HP Melmed S Genomic characterization of human and rat prolactinomas. Endocrinology (2012) 153(8):3679–91. 10.1210/en.2012-1056 PMC3404356 22635680

[65] Yu SY Hong LC Feng J Wu YT Zhang YZ Integrative proteomics and transcriptomics identify novel invasive-related biomarkers of non-functioning pituitary adenomas. Tumour Biol (2016) 37(7):8923–30. 10.1007/s13277-015-4767-2 26753958

[66] Michaelis KA Knox AJ Xu M Kiseljak-Vassiliades K Edwards MG Geraci M Identification of growth arrest and DNA-damage-inducible gene beta (GADD45beta) as a novel tumor suppressor in pituitary gonadotrope tumors. Endocrinology (2011) 152(10):3603–13. 10.1210/en.2011-0109 PMC4714647 21810943

[67] Yang Q Wang Y Zhang S Tang J Li F Yin J Biomarker Discovery for Immunotherapy of Pituitary Adenomas: Enhanced Robustness and Prediction Ability by Modern Computational Tools. Int J Mol Sci (2019) 20(1):151. 10.3390/ijms20010151 PMC6337483 30609812

[68] Kasuki L Wildemberg LE Gadelha MR MANAGEMENT OF ENDOCRINE DISEASE: Personalized medicine in the treatment of acromegaly. Eur J Endocrinol (2018) 178(3):R89–R100. 10.1530/EJE-17-1006 29339530

[69] Lines KE Stevenson M Thakker RV Animal models of pituitary neoplasia. Mol Cell Endocrinol (2016) 421:68–81. 10.1016/j.mce.2015.08.024 26320859 PMC4721536

[70] Cristina C Luque GM Demarchi G Lopez Vicchi F Zubeldia-Brenner L Perez Millan MI Angiogenesis in pituitary adenomas: human studies and new mutant mouse models. Int J Endocrinol (2014) 2014:608497. 10.1155/2014/608497 25505910 PMC4251882

[71] Karamboulas C Meens J Ailles L Establishment and Use of Patient-Derived Xenograft Models for Drug Testing in Head and Neck Squamous Cell Carcinoma. STAR Protoc (2020) 1(1):100024. 10.1016/j.xpro.2020.100024 33111077 PMC7580210

[72] Nivolumab and Ipilimumab in People With Aggressive Pituitary Tumors. Available at: https://ClinicalTrials.gov/show/NCT04042753 (Accessed September 20, 2020).

[73] Nivolumab and Ipilimumab in Treating Patients With Rare Tumors. Available at: https://ClinicalTrials.gov/show/NCT02834013 (Accessed September 20, 2020).

